# Investigating the association between metabolic syndrome conditions and perinatal mental illness: a national administrative claims study

**DOI:** 10.1186/s12884-024-06542-8

**Published:** 2024-06-07

**Authors:** Karishma Chhabria, Sudhakar Selvaraj, Jerrie Refuerzo, Chau Truong, Cecilia Ganduglia Cazaban

**Affiliations:** 1 Division of Management Policy and Community Health, Center for Healthcare Data Research, The University of Texas Health Science Center at Houston School of Public Health, Houston, TX USA; 2 Department of Public Health, Usha Kundu MD College of Health, University of West Florida 11000 University Pkwy, Pensacola, FL 32514 USA; 3 Louis Faillace, MD, Department of Psychiatry and Behavioral Sciences, University of Texas Health Science Center at Houston, McGovern Medical School, Houston, TX USA; 4 Division of Maternal-Fetal Medicine, Department of Obstetrics, Gynecology, and Reproductive Sciences, McGovern Medical School, University of Texas Health Science Center at Houston, Houston, TX USA; 5 Clinical Development, Intra-Cellular Therapies, Inc., 430 East 29th Street, New York, NY United States

**Keywords:** Perinatal comorbidities, Maternal mental health, Administrative claims data, Metabolic syndrome

## Abstract

**Background:**

Although the association between mental disorder and metabolic syndrome as a bidirectional relationship has been demonstrated, there is little knowledge of the cumulative and individual effect of these conditions on peripartum mental health. This study aims to investigate the association between metabolic syndrome conditions (MetS-C) and maternal mental illness in the perinatal period, while exploring time to incident mental disorder diagnosis in postpartum women.

**Methods:**

This observational study identified perinatal women continuously enrolled 1 year prior to and 1 year post-delivery using Optum’s de-identified Clinformatics® Data Mart Database (CDM) from 2014 to 2019 with MetS-C i.e. obesity, diabetes, high blood pressure, high triglycerides, or low HDL (1-year prior to delivery); perinatal comorbidities (9-months prior to and 4-month postpartum); and mental disorder (1-year prior to and 1-year post-delivery). Additionally, demographics and the number of days until mental disorder diagnosis were evaluated in this cohort. The analysis included descriptive statistics and multivariable logistic regression. MetS-C, perinatal comorbidities, and mental disorder were assessed using the International Classification of Diseases, Ninth, and Tenth Revision diagnosis codes.

**Results:**

372,895 deliveries met inclusion/exclusion criteria. The prevalence of MetS-C was 13.43%. Multivariable logistic regression revealed prenatal prevalence (1.64, CI = 1.59–1.70) and postpartum incident (1.30, CI = 1.25–1.34) diagnosis of mental health disorder were significantly higher in those with at least one MetS-C. Further, the adjusted odds of having postpartum incident mental illness were 1.51 times higher (CI = 1.39–1.66) in those with 2 MetS-C and 2.12 times higher (CI = 1.21–4.01) in those with 3 or more MetS-C. Young women (under the age of 18 years) were more likely to have an incident mental health diagnosis as opposed to other age groups. Lastly, time from hospital discharge to incident mental disorder diagnosis revealed an average of 157 days (SD = 103 days).

**Conclusion:**

The risk of mental disorder (both prenatal and incident) has a significant association with MetS-C. An incremental relationship between incident mental illness diagnosis and the number of MetS-C, a significant association with younger mothers along with a relatively long period of diagnosis mental illness highlights the need for more screening and treatment during pregnancy and postpartum.

## Background

Metabolic syndrome (MetS) is a global public health issue characterized by cardiovascular risk factors [1–3]. MetS are diagnosed if one has 3 or more of the following conditions, obesity, diabetes, hypertension, low HDL or high LDL (also referred here to as conditions related to MetS i.e. MetS-C). The risk of cardiovascular disease increases with each one of these conditions [4]. The Centers for Disease Control (CDC) estimates the prevalence of MetS in the USA to be around 30%, with women more likely to be diagnosed [5]. MetS in pregnant women are associated with an increased probability of developing pregnancy complications such as preterm birth, and pre-eclampsia [6]. Such outcomes place women at high risk for cardiovascular and metabolic diseases in the perinatal period (i.e. 1-year pre-delivery and up to 1 year after delivery) and later in life [6, 7]. Further, children born to women who are diagnosed with MetS are predisposed to developing MetS and mental health diseases later in life [8, 9]. 

While physical and mental health has a synergistic effect on overall health, mental illness in perinatal women is a significant complication of pregnancy and the postpartum period [10]. Untreated mental illness is associated with negative birth outcomes, such as delivering preterm or low birth weight infants thus impacting the health of children and increasing stressors in new women [11, 12]. In rare and unfortunate circumstances, untreated mental illness can lead to suicidal behaviors, with suicide being the leading cause of maternal morbidity [13, 14]. Mental illness, although common in postpartum women, is often underestimated as it parallels with symptoms characterized by a postpartum period such as fatigue and insomnia [10]. Maternal mental health has significant consequences on their own physical health and also adversely impacts their children’s physical and mental health [8, 11, 15, 16]. 

Although the association between mental disorder and MetS as a bidirectional relationship has been demonstrated [17], there is little knowledge of the effect of these conditions on peripartum mental health. Most literature focuses on maternal obesity and its impact on offspring mental illness [18, 19]. A few studies have investigated the association between diabetes and mental disorder in this population, however those studies used patient reported outcomes instead of medical diagnosis by healthcare providers or focused on low income women [20–23]. To our knowledge, no study has investigated the impact of MetS conditions (MetS-C) (any condition falling within the metabolic syndrome umbrella) and its impact on maternal mental health during the perinatal period in a national commercial claims dataset. Further, although risk of mental disorder in early perinatal period is well known, few studies have investigated the time to mental illness diagnosis in a large dataset using healthcare provider encounters [24, 25]. Understanding the role of MetS-C in a period of life already at risk for mental disorder along with evaluating healthcare encounters for mental disorder in the postpartum period is key to better planning healthcare for pregnant and postpartum women.

This study aimed to examine the prevalence rate of MetS and MetS-C in perinatal women, and its associations with mental illness. This study also aimed to descriptively explore time to mental disorder diagnosis via healthcare encounters. We hypothesized that MetS-C would be associated with an increased risk of incident mental illness in postpartum women and would be highly prevalent within 3 months postpartum.

## Methods

We followed the Strengthening the Reporting of Observational Studies in Epidemiology (STROBE) guidelines [26]. 

### Study design and population

We performed an observational study using Optum’s de-identified Clinformatics® Data Mart Database (CDM) to follow women who delivered between January 1st, 2014 - December 31st, 2019. There were 781,677 pregnancies recorded in CDM from 2014 to 2019. Of those, there were 303,703 women and 372,895 deliveries that complied with our inclusion and exclusion criteria and were included in our study. Majority of our sample included women with one delivery (*n* = 239,090, 78.72%). 19.83% of women in our sample had two deliveries and 1.45% women had 3 or more deliveries. The study cohort was defined as women (14–54 years of age) who had a hospital admission for labor and delivery using the International Classification of Disease codes (ICD-9/10) and procedure codes (see Chhabria et al. for detailed codes) [27] (Chhabria K, Sudhakar S, Refuerzo J, Ganduglia Cazaban C: Risk of postpartum psychosis in women with obstetric complications: a population cohort based registry study. Forthcoming). Only those women with continuous enrollment one-year prior to and 1-year postpartum, with no more than 90 days of an enrollment gap, were included in the study. There were no exclusion criteria for this study. CDM is derived from a database of administrative health claims for members of large commercial and Medicare Advantage health plans. CDM utilizes medical and pharmacy claims to derive patient-level enrollment information, health care costs, and resource utilization information. The population is geographically diverse, spanning all 50 states and is statistically de-identified under the Expert Determination method consistent with HIPAA and managed according to Optum® customer data use agreements 1,2. CDM administrative claims submitted for payment by providers and pharmacies are verified, adjudicated and de-identified prior to inclusion. This dataset was accessed through the University of Texas (UTHSC-H) School of Public Health Center for Health Care Data (CHCD). The study was reviewed and approved by the University of Texas Health Science Center at Houston institution’s review board (protocol number HSC-SPH-20-0073). The need for written informed consent was waived by the University of Texas Health Science Center at Houston ethics committee due to the retrospective nature of the study. All methods were carried out in accordance with relevant guidelines and regulations.

### Measurements

Our principal outcome, presence of a mental illness, was assessed using ICD-9/10 diagnostic codes (Appendix A) that capture common mental illnesses such as Depression, Anxiety, Bipolar and Psychosis during either inpatient or outpatient care. Diagnosis of mental illness was categorized based on when diagnosis occurred, i.e., within one year prior to delivery was considered “pre-delivery,” and the year following delivery was considered “post-delivery.” Incident diagnosis were those that occurred in the post-delivery phase for which there was no evidence of mental illness in the year prior to delivery.

Presence of MetS related conditions were assessed using ICD-9/10 diagnosis codes (Appendix B) for obesity, diabetes, hypertension, low HDL or high LDL in medical claims data for the year prior to delivery. The presence of three or more of these conditions or a specific diagnosis code for metabolic syndrome was used to classify individuals as having MetS. The ICD codes for MetS-C are distinct from similar conditions occurring during pregnancy. For instance, diabetes mellitus has a different ICD code than gestational diabetes.

Other co-variates included other perinatal comorbidities such as gestational hypertension, placenta previa, pre-eclampsia, severe eclampsia, gestational diabetes and having a previous cesarean. These were assessed using ICD-9/10 diagnosis codes present anytime 9 months prior to delivery and up to 4 months after delivery (Appendix C). Additionally, age at delivery (categorized as 18 years and younger, 19–34 years and 35 years and older) and race/ethnicity as recorded in the patient’s enrollment file were evaluated.

### Statistical analysis

We performed descriptive statistics on deliveries, evaluating means, standard deviations, prevalence, and incidence as appropriate. The unit of analysis was the specific delivery. Bivariate analysis included chi-square and t-test models. Unadjusted, adjusted odds ratios (OR) and confidence intervals (CI) were calculated using mental illness as a binary variable reflecting the presence or absence of a diagnosis. MetS-C diagnosis was calculated as a 4-level categorical variable reflecting no, one, two or three and more MetS-C. Multivariable logistic regression models were used to evaluate the risk of mental illness based on MetS-C diagnosis, presence or absence of any perinatal conditions, age at delivery, and race/ethnicity. We also evaluated the number of days to mental illness diagnosis after delivery for postnatal incident diagnosis. Statistical significance was set at 0.05 apriori. The goodness of fit statistics and model convergence status were evaluated to assess model significance. Statistical analysis was performed using SAS, version 9.4 (SAS Institute Inc).

## Results

Women were primarily White (63.91%), with a mean age at the time of delivery of 31.86 years (SD = 5.26 years). MetS-C (one or more of the listed conditions) were diagnosed in 50,081 (13.43%) deliveries within 1 year prior to delivery. Of these, obesity (9.95%) and high blood pressure (2.91%) were the most commonly occurring conditions. Prevalence of MetS in our cohort (as defined as having 3 or more MetS-C) was 1.80% (897) deliveries. Perinatal comorbidities were present in 42.85% of deliveries, gestational diabetes (18.35%) and gestational hypertension (9.21%) being the most common. Among women diagnosed with at least one MetS-C, other perinatal comorbidities were significantly more likely (54.86%) to be diagnosed than among those with not MetS related conditions. Table 1 presents descriptive statistics for deliveries by women with and without MetS-C.

**Table 1:** Demographic Characteristics of Deliveries Included in the Study by Exposure

	Deliveries with MetS-C *n* (%)	Deliveries without MetS-C *n* (%)	*P*-value
N (%)	50,081 (13.43)	322,814 (86.57)	
Age (mean, SD)	32.32 (SD = 5.38)	31.41 (SD = 5.13)	< 0.001
Race/ethnicity			
White	30,455 (60.81)	216,304 (67.01)	< 0.001
Black	6,645 (13.27)	24,764 (7.67)	< 0.001
Hispanic	8,277 (16.53)	41,322 (12.80)	< 0.001
Asian	2,853 (5.70)	28,120 (8.71)	< 0.001
Missing	1,851 (3.70)	12,304 (3.81)	
Perinatal comorbidity	27,476 (54.86)	131,670 (40.79)	< 0.001
Number of MetS-C			
One	43,542 (86.94)		
Two	5,642 (11.27)		
Three or more	897 (1.79)		
Type of MetS-C			
Obesity	37,059 (73.99)		
Diabetes	2,755 (5.50)		
High blood pressure	10,854 (21.67)		
Low HDL	113 (0.23)		
Triglycerides	6,759 (13.50)		

MetS-C: Metabolic Syndrome Conditions

SD: Standard Deviation

A total of 15,457 deliveries (18.61%) had a mental illness diagnosis. Of these, 10,091 (65.28%) deliveries had evidence of a mental illness condition in the year prior to delivery (p-value < 0.001). Depression and anxiety were the most prevalent mental illness among those with and without MetS-C. Although statistically significant in both categories (*p* < 0.05), the prevalence of depression and anxiety were much higher in those with MetS-C (10.45% and 15.35%, respectively) as compared to those without MetS-C (5.44% and 9.16%, respectively). Among the deliveries who had a post-delivery diagnosis of mental illness, 6,991 (13.96%) deliveries were identified as having incident diagnosis of mental illness i.e. they did not have a documented history of mental illness within the year prior to delivery. Breakdown of the prevalence of the type of mental illness is shown in Table 2.

**Table 2:** Prevalence and Incidence of Mental Illness Stratified by Perinatal Period

*N* (%)	Prenatal diagnosis	Postnatal diagnosis	Incident diagnosis
With MetS-C	10,091 (20.15%)	11,663 (23.29%)	6,991 (13.96%)
Depression	5,233 (10.45%)	6,968 (13.91%)	3,891 (7.77%)
Bipolar	891 (1.78%)	941 (1.88%)	368 (0.73%)
Anxiety	7,689 (15.35%)	7,948 (15.87%)	4,264 (8.51%)
Psychosis	123 (0.25%)	117 (0.23%)	86 (0.17%)
Without MetS-C	38,313 (11.87%)	48,901 (15.15%)	34,710 (10.75%)
Depression	17,527 (5.44%)	26,517 (8.21%)	17,569 (5.44%)
Bipolar	2,453 (0.76%)	3,032 (0.94%)	1,637 (0.51%)
Anxiety	29,575 (9.16%)	32,740 (10.14%)	22,967 (7.11%)
Psychosis	294 (0.09%)	285 (0.09%)	310 (0.10%)

MetS-C: Metabolic Syndrome Conditions

Unadjusted and adjusted results for prenatal and postnatal incident mental illness are shown in Table 3. Unadjusted analysis showed that having one MetS-C significantly increased the odds of having a mental disorder diagnosis in the prenatal period (1.69, CI = 1.62–1.74) and an incident postpartum diagnosis (1.32, CI = 1.27–1.37). Further, unadjusted odds of being diagnosed with postnatal incident mental illness increased with an increasing number of MetS-C (2 MetS-C 1.57, CI = 1.43–1.71; 3 or more MetS-C 2.13, CI = 1.12–4.02). The unadjusted presence of perinatal comorbidities was also significantly associated with a higher risk of mental illness for both prenatal (1.37 CI = 1.28–1.35) and postpartum incident diagnosis (1.25 CI = 1.22–1.29). Caucasian women were more likely to be diagnosed with a mental illness as compared to other races.

**Table 3:** Unadjusted and Adjusted Odds Ratio for Prenatal and Incident Mental Illness

Prenatal Mental Health	Unadjusted OR (CI)	Adjusted OR (CI)
MetS-C		
One MetS-C	1.69 (1.62–1.74) *	1.64 (1.59–1.70) *
2 MetS-C	2.83 (2.63–3.04) *	2.68 (2.49–2.88) *
3 or more MetS-C	1.77 (0.88–3.53)	1.64 (0.82–3.30)
Perinatal comorbidities	1.37 (1.28–1.35) *	1.25 (1.22–1.29) *
Age		
18 years and younger	1.89 (1.65–2.16) *	2.00 (1.75–2.28) *
35 years and older	1.12 (1.09–1.15) *	1.09 (1.06–1.12) *
Race/Ethnicity		
Asian	0.36 (0.34–0.38) *	0.37 (0.35–0.39) *
Black	0.79 (0.75–0.83) *	0.73 (0.69–0.76) *
Hispanic	0.70 (0.67–0.73) *	0.67 (0.64–0.70) *
**Incident Mental Health**	Unadjusted OR	Adjusted OR (CI)
MetS-C		
One MetS	1.32 (1.27–1.37) *	1.30 (1.25–1.34) *
2 MetS-C	1.57 (1.43–1.71) *	1.51 (1.39–1.66) *
3 or more MetS-C	2.13 (1.12–4.02) *	2.12 (1.21–4.01) *
Perinatal comorbidities	1.27 (1.24–1.31) *	1.26 (1.23–1.29) *
Age		
18 years and younger	1.67 (1.46–1.91) *	1.70 (1.49–1.95) *
35 years and older	0.89 (0.87–0.91) *	0.88 (0.86–0.91) *
Race/Ethnicity		
Asian	0.43 (0.41–0.46) *	0.44 (0.41–0.47) *
Black	0.84 (0.80–0.88) *	0.81 (0.77–0.84) *
Hispanic	0.77 (0.74–0.80) *	0.75 (0.72–0.78) *

Prenatal mental illness diagnosed 1 year prior to delivery

Incident mental illness diagnosed during 1 year post-delivery with no history of mental illness in the 1 year prior to delivery

Mental illness diagnosis included depression, anxiety, bipolar and psychosis

MetS-C: Metabolic Syndrome Conditions

SD: Standard Deviation

*: *p* < 0.05

Multivariable logistic regression met the criteria for convergence and goodness of fit. The adjusted OR of being diagnosed with a mental illness in the prenatal period was 1.64 times higher (CI = 1.59–1.70) for those with one MetS-C and 2.68 times higher (CI = 2.49–2.88) for those with two MetS-C as compared to those without MetS-C after adjusting for presence of a perinatal co-morbidity (1.25 CI = 1.22–1.29), age and race. Women younger than 18 years of age were twice as more likely to be diagnosed with prenatal mental disorder as compared to older women (CI = 1.75–2.28). The adjusted OR of being diagnosed with an incident mental illness in the postnatal period was 1.30 times higher (CI = 1.25–1.34) for those with one MetS-C, 1.51 times higher (CI = 1.39–1.66) for those with two MetS-C and 2.12 times higher (CI = 1.21–4.01) for those with 3 or more MetS-C as compared to those without MetS-C after adjustment for covariates. The presence of perinatal co-morbidity was a statistically significant risk factor (1.26 CI = 1.23–1.29). Further, women under the age of 18 years were 1.7 times more likely to have an incident mental disorder diagnosis in the postnatal period as compared to women of other age groups. After adjusting for all covariates included in our models, OR for having a prenatal mental health diagnosis and incident postnatal mental health diagnosis was incremental in nature for one MetS-C and two MetS-C, even after excluding obesity (Appendix D).

We also evaluated time to incident mental illness diagnosis during the postpartum period measuring the number of days from hospital discharge after delivery to first provider encounter where a diagnosis of mental illness was recorded (Table 4). On average, it took 157 days (SD = 103 days) for an incident mental illness diagnosis in the post-partum period to be recorded. Specifically, anxiety and bipolar disorders (median > 160 days, IQR = 174 days) took the longest to diagnose, followed by depression (median = 130 days, IQR = 171 days) and psychosis (median = 110 days, IQR = 176 days). Presence of MetS-C or perinatal comorbidities did not show an association with earlier diagnosis of mental illness.

**Table 4:** Number of Days to Mental Health Diagnosis

Median (IQR)	Incident diagnosis
Overall	
Depression	130 (171)
Anxiety	165 (174)
Bipolar	163 (174)
Psychosis	110 (176)
**With MetS-C**	
Depression	120 (169)
Anxiety	149 (170)
Bipolar	162 (185)
Psychosis	101 (186)
**With Perinatal Comorbidities**	
Depression	122 (171)
Anxiety	160 (176)
Bipolar	156 (179)
Psychosis	117 (195)

MetS-C: Metabolic Syndrome Conditions

IQR: Interquartile Range

## Discussion

### Main findings

This study investigated the risk of MetS via any of the conditions associated with MetS and perinatal mental illness. The primary analysis found an increased risk of mental illness in women during both prenatal and postpartum periods, with any one of the MetS-C. In addition, being diagnosed with two or more MetS-C had a significantly elevated risk of mental illness. Our results show a positive association with increasing number of MetS-C to increasing odds of being diagnosed with a mental illness during prenatal and postpartum periods. Furthermore, younger women (18 years and younger) were at significant risk of mental illness in the prenatal and postnatal periods. Most of these cases were diagnosed with depression and anxiety followed by bipolar disorder and psychosis. Lastly, our study highlighted the long length of time to incident mental illness diagnosis in these women.

### Strengths and limitations

The strengths of this study include the use of a database, which covers a large population, allowing the assessment of less frequent events. Our study included data from 2014 to 2019 to improve our knowledge on more recent trends. However, the study is limited by lack of more detailed clinical information (such as laboratory values and history of medication usage/adherence) along with socioeconomic factors (such as marital status and household income) which could have shed more light on the effect of severity of MetS-C on mental illness. The study used a commercial insurance database which lacks information on patient socioeconomic status which underpins the physical-mental relationship, especially in the perinatal period. Moreover, due to the nature of the data, which is created through billing for health care, we are limited to assessing disease when health care encounters occur. However, it is less likely a mother would undergo a mental health event and have no encounter with a healthcare provider. The data is ideally suited to capture all encounters regardless of provider affiliation and network. Although ICD diagnosis codes clearly state the difference between gestational, type 1 and type 2 diabetes and that our study did not include gestational diabetes (GDM) codes to classify MeTS-C, it could be possible that some providers may have incorrectly coded GDM as type 2 diabetes. Our analysis considered the most serious (psychosis) and most commonly occurring mental illness (anxiety and depression) in these women. However, we did not include other mental illnesses such as substance use disorder, post-traumatic stress disorder, neurodevelopmental conditions etc. Further, we had 1,864 (0.49%) women who had 2 or more pregnancies with MetS-C being reported in first pregnancy. Since these conditions are altered by lifestyle changes, we did not recode the subsequent pregnancies as having MetS-C. Last, insurance claims data is always at risk of miss or upcoding diagnosis i.e. reporting of more serious diagnosis/procedures to insurance companies than they actually performed. This study though used standardized and published algorithms used on similar databases to measure disease-related conditions [28] (Chhabria K, Sudhakar S, Refuerzo J, Ganduglia Cazaban C: Risk of postpartum psychosis in women with obstetric complications: a population cohort based registry study. Forthcoming). 

### Interpretation

There is a growing body of evidence linking maternal obesity and offspring neurological and psychiatric disorders due to various biological pathways such as dysregulated serotonergic and dopaminergic signaling. Studies have shown a link between abnormal dopamine signaling and serotonin deficiency with major depressive disorder [29–31]. This relationship could explain the association between metabolic syndrome and mental illness in perinatal women. It is however most likely that biopsychosocial mechanism may play a role. For example, MetS-C are associated with other psychosocial adversities; early life trauma, anxiety or depression, and bullying can cause behavioral problems, unhealthy lifestyles that increases the chance of metabolic syndrome [32]. 

A recent literature review investigating the association on maternal obesity and perinatal depression found conflicting results in 7 studies they evaluated [33]. Our study helps shed light on the association of obesity and other metabolic syndrome conditions with maternal mental health. Further, most of the studies in the literature review used patient reported outcomes to measure mental health [34, 35]. Our study is different as we investigated confirmed diagnoses of mental illness within a larger sample size. Furthermore, our study findings are in line with another recent study investigating maternal obesity and mood disorders [35]. Similar to our findings, they found a significant association between obesity and maternal mental health in the prenatal phase as compared to non-obese prenatal women (30.4% versus 21.2%, *p* < 0.01) [35]. 

Evidence suggests that metabolic conditions are often associated with elevated systemic inflammation which can be a common risk factor for mental illnesses [36–38]. Studies have shown a bidirectional relationship between MetS-C and mental illness [36–38]. It links dysregulated inflammatory markers, such as cortisol and C-reactive protein, which are known for glucose and lipid metabolism, with poorer health outcomes in those with mental illness [36–38]. Implications of this highlights the need to identify those with MetS for efficacious treatment modalities for mental health [36–38]. Translating these findings to perinatal women, our study supports the overall findings, indicating MetS-C during pregnancy is associated with the risk of perinatal complications which can predispose to postpartum mental illnesses.

Our study highlights the need for targeted screening and intervention for women with high risk of mental health problems during pregnancy, especially younger women. Appropriately addressing mental health issues during pregnancy can reduce maternal and child complications [39]. Although there is an abundance of literature regarding obesity, diabetes, and hypertension in pregnancy leading to an increased risk of perinatal morbidity, there are few articles focusing particularly on MetS in pregnancy. MetS in pregnancy increases the risk of preeclampsia 2 to 5-fold [7, 40]. There is some evidence to suggest that MetS increases the risk of preterm labor [41]. However, the greatest pregnancy risk from MetS is gestational diabetes [7, 42–45]. Moreover, MetS noted in the postpartum period and in women with a history of GDM predisposed them to Type 2 Diabetes later in life [46]. Another recent investigation showed a significant association between severe maternal morbidity and mental illness in perinatal women (Chhabria K, Sudhakar S, Refuerzo J, Ganduglia Cazaban C: Risk of postpartum psychosis in women with obstetric complications: a population cohort based registry study. Forthcoming). Similar to our findings, these studies highlight the association of metabolic syndrome leading to mental illness.

## Conclusion

Our study highlights the need for targeted screening and intervention for women, especially younger women, with a high risk of mental health problems during pregnancy and postpartum as recommended by the American College of Obstetricians and Gynecologists (ACOG) [47]. Among this perinatal population, screening for depression itself resulted in clinical benefits resulting in an absolute reduction of depression by 2.1 to 9.1% [48]. Future studies should consider developing a risk calculator model for pregnant women to evaluate risk of mental illness in patients with MetS-C. This would help create targeted interventions to promote healthy living as primary prevention steps. Future studies should also include information for education and physical activity as well as social determinants of health, which are known to be associated with an increase the risk of MetS [49]. Future studies should also assess the association between MetS conditions and individual mental health disorders and the effect of antipsychotics medications on this association. An interesting finding of our study is the relatively long time period of diagnosis and treatment for mental illness in the postpartum period within our database which ranged from 110 to 174 median days. ACOG suggests that postpartum women should be seen by their obstetrician-gynecologist or other obstetric care providers within the first 3 weeks postpartum, followed up no later than 12 weeks after birth [50]. However, our study shows that this is not a sufficient length of time to follow these postpartum women who present with mental illness on average at 157 days, or approximately 22 weeks after delivery, with a range of approximately 3.7 to 5.8 months. More likely than not, other primary care providers than the obstetrician-gynecologist are making these diagnoses and proceeding with much needed treatment. Efforts should be made to extend the recommended length of the postpartum period to 6 months or even a year after delivery. As importantly is the need to educate all primary care providers to be aware of the presentation of mental illness related to pregnancy and the postpartum period to extend several months postpartum.

Understanding the mechanisms through which MetS as well as perinatal co-morbidities, demographic and social factors affect the risk of suffering postpartum mental health issues is key to finding the most efficient treatment and prevention strategies.

## **Appendix A:** Codes for capturing mental illness

**Table:** 

	ICD − 9	ICD-10
Depression	296.2x, 296.3x, 300.4x, 301.12, 311x, 648.42, 648.44	F32x, F33x, F34.1x, F34.8x, F34.9x, F53.0x, O90.6x
Anxiety	300.0x, 300.2x,	F40x, F41x
Bipolar	296.0x, 296.14, 296.23, 296.34, 296.4x, 296.5x, 296.6x, 296.7x, 296.8x, 296.9x	F30x, F31x, F34.0x, F39x
Psychosis	295x, 297x	F20x – F29x, F53.1x

## **Appendix B:** Codes for capturing metabolic syndrome conditions

**Table:** 

	ICD − 9	ICD-10
Obesity	278.00x, 278.01x	E66.1x, E66.2x, E66.8x, E66.9x
Diabetes	250.00, 250.02, 790.21	E11x, R73.01
High blood pressure	401x − 405x	I10x - I16x
Low HDL	272,5	E78.6x
Triglycerides	272.1x – 272.4x	E78.0x - E78.9x

## **Appendix C:** Codes for capturing perinatal comorbidities

**Table:** 

Perinatal comorbidities	ICD − 9	ICD-10
Placenta Previa	641.0x, 641.1x	O44.x
Gestational hypertension	642.3x	O13.x
Preeclampsia	642.4x, 642.7x	O14.x
Eclampsia	642.5x, 642.6x	O15.x
Gestational diabetes	648.9x	O24.41x
Previous cesarean section	654.2x	O34.21x
Preterm labor	644x	O60x

## **Appendix D:** Unadjusted and adjusted odds ratio for prenatal and incident mental illness without obesity

**Table:** 

Prenatal Mental Health	Unadjusted OR (CI)	Adjusted OR (CI)
MetS-C		
One MetS-C	2.01 (1.92–2.11) *	1.95 (1.85–2.05) *
2 MetS-C	2.62 (2.20–3.13) *	2.46 (2.15–2.82) *
3 or more MetS-C	1.67 (0.84–3.35)	1.72 (1.05–2.82) *
Perinatal comorbidities	1.32 (1.28–1.35) *	1.278 (1.25–1.31) *
Age		
18 years and younger	1.89 (1.66–2.16) *	1.94 (1.70–2.21) *
35 years and older	1.12 (1.09–1.15) *	1.09 (1.06–1.12) *
Race/Ethnicity		
Asian	0.36 (0.34–0.38) *	0.36 (0.34–0.38) *
Black	0.79 (0.75–0.82) *	0.75 (0.72–0.79) *
Hispanic	0.69 (0.67–0.72) *	0.68 (0.65–0.71) *
**Incident Mental Health**	Unadjusted OR	Adjusted OR (CI)
MetS-C		
One MetS	1.32 (1.25–1.39) *	1.31 (1.24–1.38) *
2 MetS-C	1.37 (1.09–1.71) *	1.45 (1.23–1.71) *
3 or more MetS-C	2.06 (1.09–3.90) *	1.58 (0.94–2.65)
Perinatal comorbidities	1.28 (1.24–1.31) *	1.27 (1.24–1.31) *
Age		
18 years and younger	1.67 (1.46–1.91) *	1.68 (1.46–1.91) *
35 years and older	0.89 (0.86–0.91) *	0.88 (0.86–0.91) *
Race/Ethnicity		
Asian	0.43 (0.41–0.46) *	0.43 (0.41–0.46) *
Black	0.84 (0.80–0.88) *	0.82 (0.78–0.86) *
Hispanic	0.76 (0.73–0.79) *	0.76 (0.73–0.79) *

Prenatal mental illness diagnosed 1 year prior to delivery

Incident mental illness diagnosed during 1 year post-delivery with no history of mental illness in the 1 year prior to delivery

Mental Illness diagnosis included depression, anxiety, bipolar and psychosis

*MetS-C: Metabolic Syndrome Conditions

SD: Standard Deviation

*: *p* < 0.05

## Data Availability

The datasets generated and/or analyzed during the current study are not publicly available as they are confidential information but are available from CDM reasonable request. CDM may be contacted for the data at optum.com.

## Abbreviations

ACOG: American College of Obstetricians and Gynecologists
CDM: Optum’s de-identified Clinformatics® Data Mart Database
CI: Confidence Interval
GDM: Gestational Diabetes Mellites
ICD: International Classification of Diseases
IQR: Interquartile Range
MetS: Metabolic Syndrome
MetS-C: Metabolic Syndrome Conditions
OR: Odds Ratio
